# Smooth, an hnRNP-L Homolog, Might Decrease Mitochondrial Metabolism by Post-Transcriptional Regulation of Isocitrate Dehydrogenase (Idh) and Other Metabolic Genes in the Sub-Acute Phase of Traumatic Brain Injury

**DOI:** 10.3389/fgene.2017.00175

**Published:** 2017-11-15

**Authors:** Arko Sen, Katherine Gurdziel, Jenney Liu, Wen Qu, Oluwademi O. Nuga, Rayanne B. Burl, Maik Hüttemann, Roger Pique-Regi, Douglas. M. Ruden

**Affiliations:** ^1^Institute of Environmental Health Sciences, Wayne State University, Detroit, MI, United States; ^2^Department of Pharmacology, Wayne State University, Detroit, MI, United States; ^3^C. S. Mott Center for Human Growth and Development, Department of Obstetrics and Gynecology, Wayne State University, Detroit, MI, United States; ^4^Center for Molecular Medicine and Genetics, Wayne State University, Detroit, MI, United States; ^5^Department of Obstetrics and Gynecology, Wayne State University, Detroit, MI, United States

**Keywords:** traumatic brain injury, alternative splicing, intron retention, histone modification, smooth, hnRNP-L

## Abstract

Traumatic brain injury (TBI) can cause persistent pathological alteration of neurons. This may lead to cognitive dysfunction, depression and increased susceptibility to life threatening diseases, such as epilepsy and Alzheimer's disease. To investigate the underlying genetic and molecular basis of TBI, we subjected *w*^1118^ *Drosophila melanogaster* to mild closed head trauma and found that mitochondrial activity is reduced in the brains of these flies 24 h after inflicting trauma. To determine the transcriptomic changes after mild TBI, we collected fly heads 24 h after inflicting trauma, and performed RNA-seq analyses. Classification of alternative splicing changes showed selective retention (RI) of long introns (>81 bps), with a mean size of ~3,000 nucleotides. Some of the genes containing RI showed a significant reduction in transcript abundance and are involved in mitochondrial metabolism such as Isocitrate dehydrogenase (Idh), which makes α-KG, a co-factor needed for both DNA and histone demethylase enzymes. The long introns are enriched in CA-rich motifs known to bind to Smooth (Sm), a heterogeneous nuclear ribonucleoprotein L (hnRNP-L) class of splicing factor, which has been shown to interact with the H3K36 histone methyltransferase, SET2, and to be involved in intron retention in human cells. H3K36me3 is a histone mark that demarcates exons in genes by interacting with the mRNA splicing machinery. Mutating sm (*sm*^4^/Df) resulted in loss of both basal and induced levels of RI in many of the same long-intron containing genes. Reducing the levels of Kdm4A, the H3K36me3 histone demethylase, also resulted in loss of basal levels of RI in many of the same long-intron containing genes. Chromatin immunoprecipitation followed by deep sequencing (ChIP-seq) for H3K36me3 revealed increased levels of this histone modification in retained introns post-trauma at CA-rich motifs. Based on these results, we propose a model in which TBI temporarily decreases mitochondrial activity in the brain 24 h after inflicting trauma, which decreases α-KG levels, and increases H3K36me3 levels and intron retention of long introns by decreasing Kdm4A activity. The consequent reduction in mature mRNA levels in metabolism genes, such as Idh, further reduces α-KG levels in a negative feedback loop. We further propose that decreasing metabolism after TBI in such a manner is a protective mechanism that gives the brain time to repair cellular damage induced by TBI.

## Introduction

Traumatic brain injury (TBI) is a complex pathological condition associated with high mortality rates (Unterharnscheidt, 1995; Ling et al., 2015). TBI can be divided into 2 major categories: severe TBI or concussive injury and mild TBI (MTBI) or sub-concussive injury (Broglio et al., 2012). Concussive injury is characterized by dramatic changes in neuronal homeostasis including rapid influx of calcium, efflux of potassium, release of neurotransmitters and widespread neuronal death (Giza and Hovda, 2014). In contrast, sub-concussive injury is subtler and reported to cause multifocal microscopic axonal damage and micro-hemorrhages (McKee and Robinson, 2014; Daneshvar et al., 2015), which may lead to progressive neurodegeneration and increased susceptibility to Alzheimer's, Parkinson's or motor neuron diseases in later life (Ling et al., 2015).

A study by the Wasserman lab reported that the fundamental characteristics of human TBI also occur in Drosophila. Drosophila inflicted with TBI with a high-impact trauma (HIT) device showed temporary incapacitation, ataxia and reduction in lifespan post-TBI. These flies developed vacuolar lesions in central regions of the brain in adult life (>14 days), indicative of progressive neurodegeneration (Katzenberger et al., 2013). Barekat and colleagues have recently shown similar results by inflicting trauma on Drosophila using a vortex mixer (Barekat et al., 2016).

Using the Drosophila model of TBI with the HIT device (Katzenberger et al., 2013), we investigated the impact of sub-concussive injury on alternative splicing and found widespread alternative splicing changes 24 h post-trauma. We found that alternative splicing changes are dominated by intron retention and might be regulated by the heterogeneous nuclear ribonucleoprotein L (hnRNP-L) class of splicing factors, which is uniquely represented by Smooth (Sm) in Drosophila. This preliminary study provides a potential novel mechanism of transcriptional regulation in the sub-acute phase of TBI and will be further explored and validated in future investigations.

## Results

### Increase in number of strikes result in progressive decrease in long-term survival

**Figure 1:**
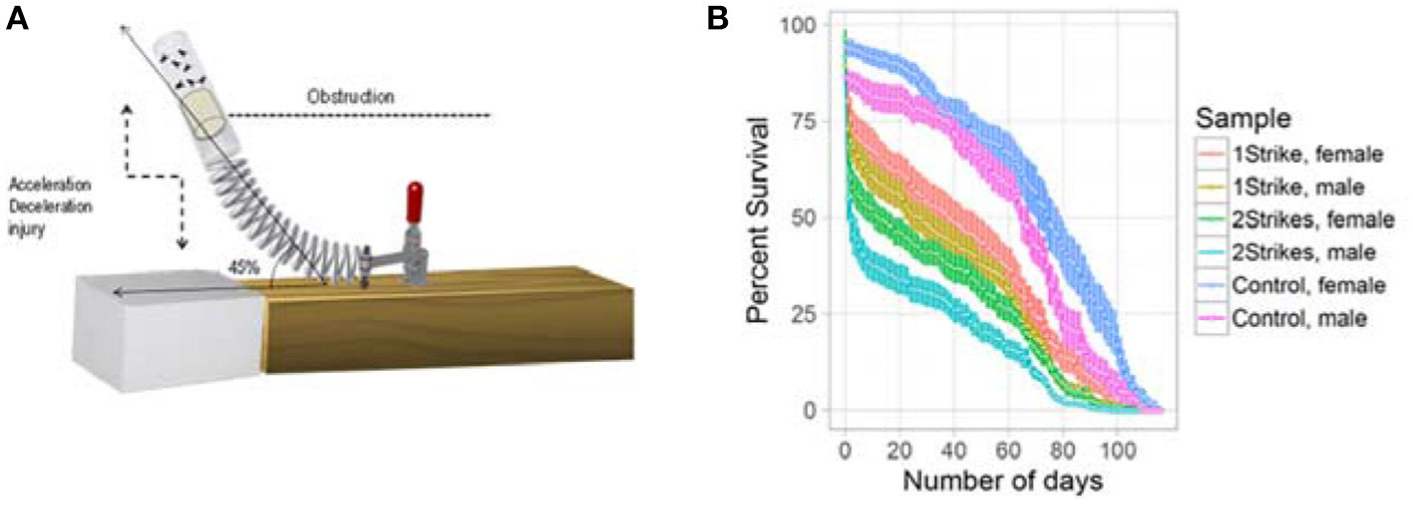
Strike-dependent decrease in life-span: **(A)** Illustration of the modified high impact trauma device (HIT). **(B)** Survival curve (mean ± standard error) of Drosophila subjected to either 1 strike or 2 strike. We used 20 *w*^1118^ flies per vial and repeated the experiment 5 times for male and female flies.

We simulated closed head trauma in Drosophila using a high impact trauma (HIT) device with a 45° spring deflection to attenuate the impact (Figure 1A). The design of our HIT device and the forces generated are described previously (Katzenberger et al., 2013). We performed survival estimation, after 1 or 2 strikes, using 20 flies per vial and repeated the experiment 5 times for male and female flies, separately. We could replicate the strike dependent decrease in survival previously observed using a 90° spring deflection (Katzenberger et al., 2013). The results as illustrated in Figure 1B confirmed that the HIT model of TBI in Drosophila is reproducible and reliable.

### Characterization of alternative splicing events post-TBI

**Figure 2:**
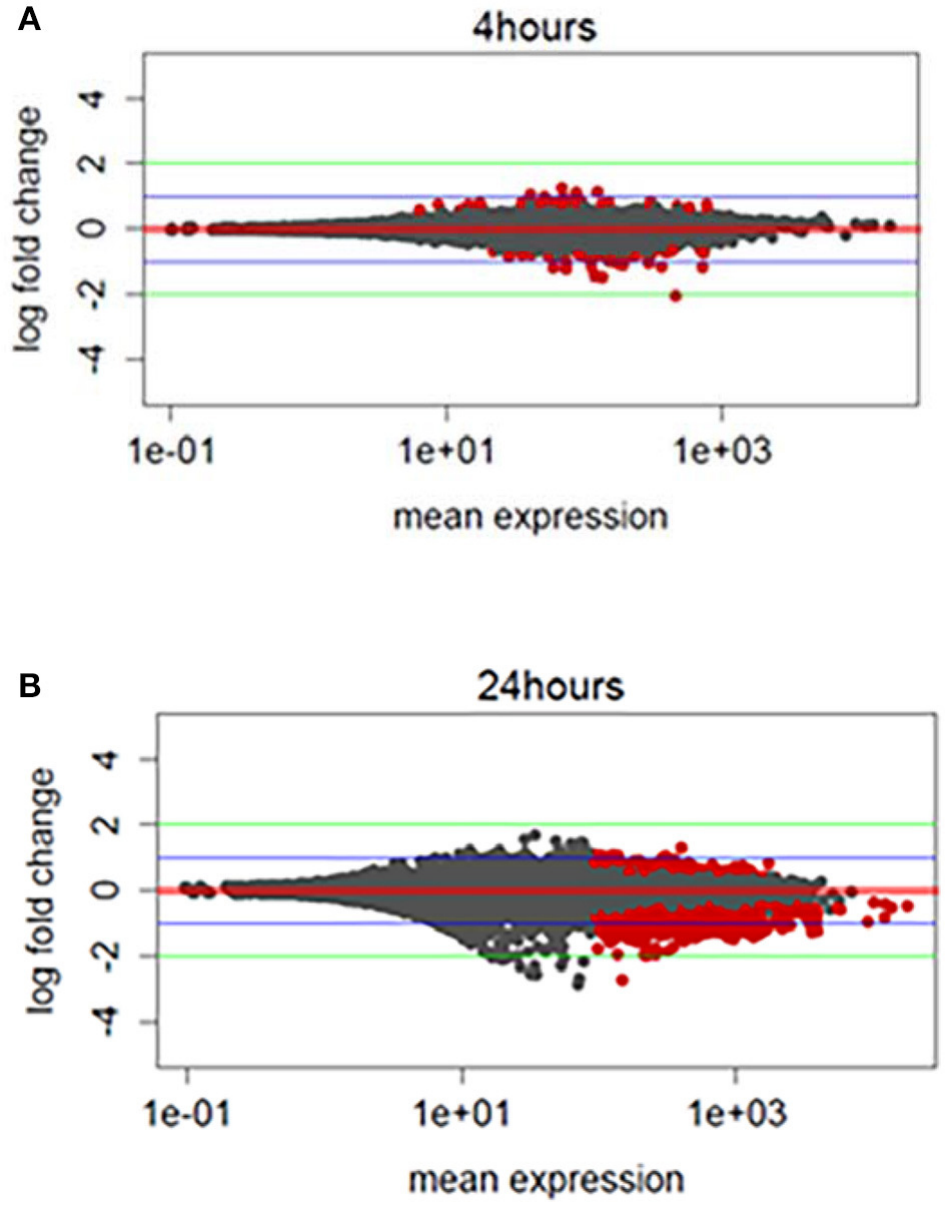
Exploratory analysis of RNA-sequencing profile: **(A)** MA (Mean expression vs. log_2_-fold change) plots differentially expressed genes for TBI heads collected 4 h post-TBI and, **(B)** 24 h post-TBI. Red dots indicate the genes associated with FDR corrected *p* ≤ 0.1 or 10%. Green horizontal line indicates a logFC ≥ ±2 and blue horizontal line indicate a logFC ≥ ±1.

We hypothesized that TBI will cause changes in gene expression in the fly brain. To study gene expression changes that result from sub-concussive injury, we performed an analysis of RNA-seq data comparing controls to heads collected 4 and 24 h after TBI separately for male and female flies (Katz et al., 2010; Figure 2). We found more differential gene expression after 24 h compared with 4 h, but the number of significant genes with gene expression changes greater than 4-fold higher or lower was very small (Figure 2, red dots above or below the green line). Consequently, we focused instead on alternative splicing events induced by TBI at 24 h, which had a much greater number of affected genes.

**Figure 3:**
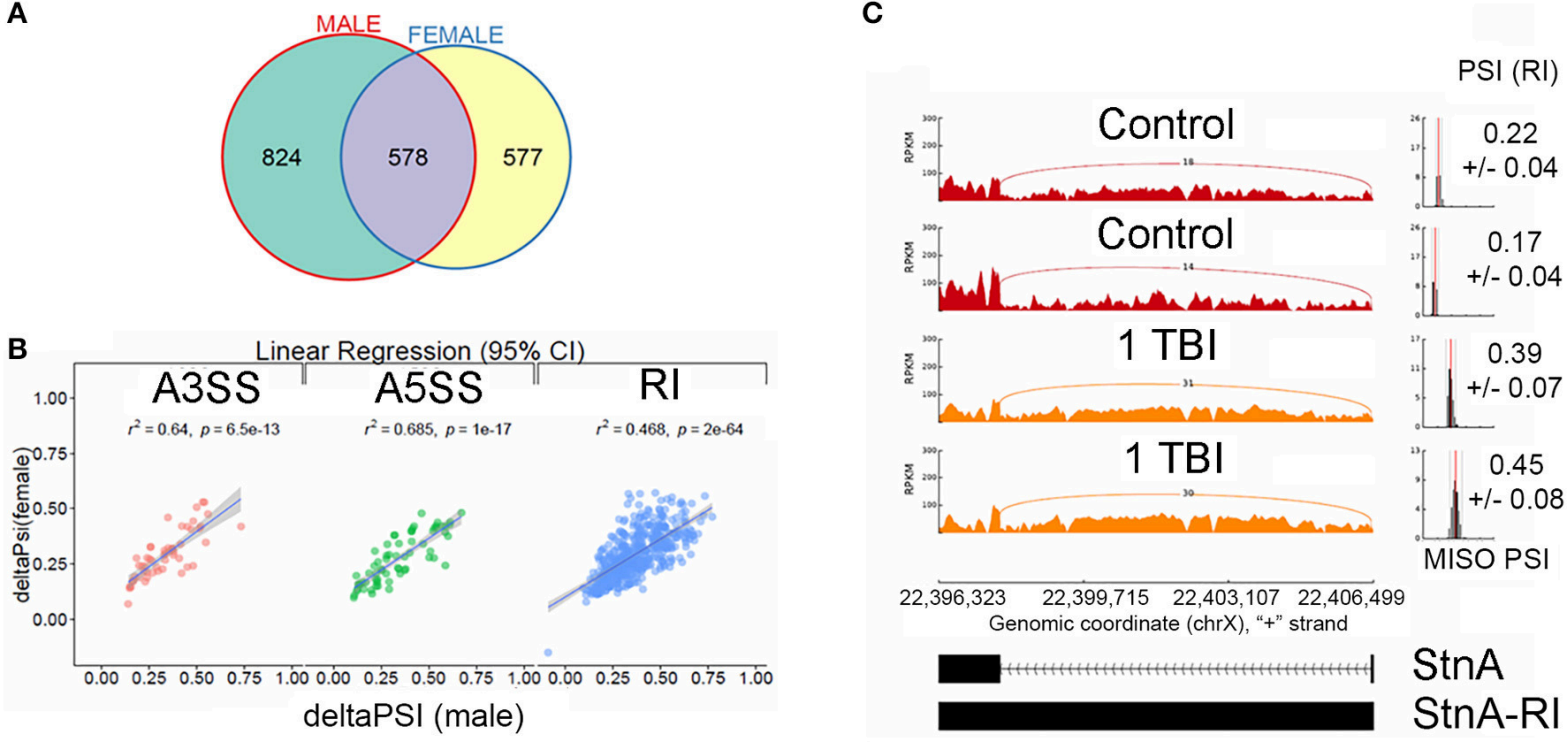
Characterization of alternative splicing events in Drosophila model of TBI: **(A)** Overlaps between statistically significant differential alternative splicing events detected using MISO for heads collected 24 h post-TBI for males and females. **(B)** Linear regression between ΔPSI or deltaPSI for differential splicing events detected for heads collected 24 h post-TBI for males and females. **(C)** Sashimi plot showing intron retention event in Stoned A (StnA). Percent spliced in (PSI) represents the fraction of the intron that is retained (RI) compared with the exon sequences present. The PSI values increased from ~20% in the controls to ~40% in the TBI (*p*-value (*t*-test) < 0.001). The data is represented as Reads Per Kilo-base per Million.

**Figure 4:**
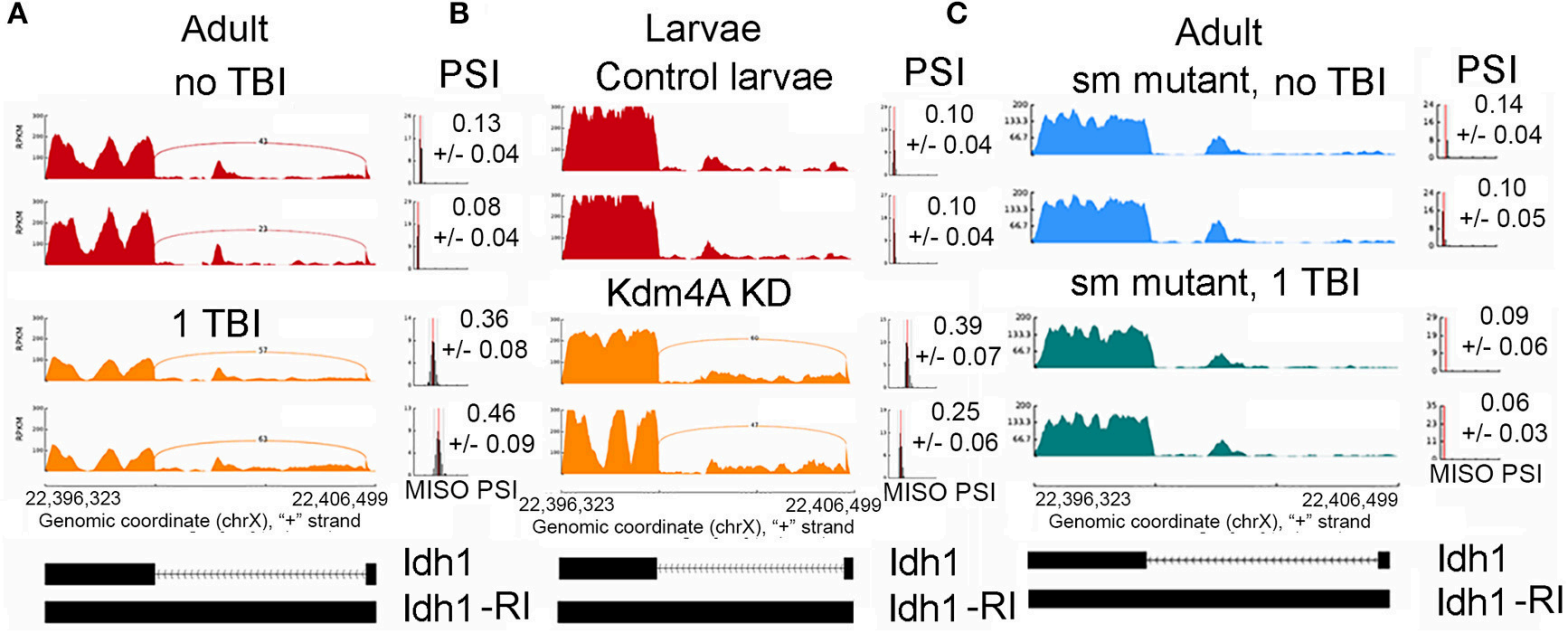
Increase in intron retention Isocitrate dehydrogenase (Idh). Sashimi plots showing splicing profile of Isocitrate dehydrogenase (Idh). **(A)**, w^1118^ male and female controls (non-TBI) heads and heads collected 24 hrs post-TBI. Notice the increase in PSI after TBI (*p* < 0.01). **(B)**, Control 3rd instar larvae and dKDM4A-mutant 3rd instar larvae. Notice the increase in PSI after TBI (*p* < 0.01). **(C)**, Comparison between male or female *sm*-mutant (±TBI) heads collected 24 h post-TBI. Notice the PSI did not significantly change after TBI (*p* = 0.76).

We filtered differential splicing events using difference in Percent Spliced In (ΔPSI) ≥ ±5% and Bayes Factor ≥10. A Bayes factor (BF) is a statistical index that quantifies the evidence for a hypothesis, compared to an alternative hypothesis. Of the 578 events, 458 events were intron retention (RI) events that were shared between males and females (Figure 3A). Mixture of Isoforms probabilistic model for RNA-seq (MISO) (Katz et al., 2010) analysis for RI events in the neurodevelopmental gene Stoned A (StnA), which has a 605 nucleotide first exon and an 8823 nucleotide first intron (Figure 3C), and the metabolic gene Isocitrate dehydrogenase (IDH), which has a 237 nucleotide first exon and a 2,887 nucleotide first intron (Figure 4A), are shown. We observed a positive correlation of 46.8% between ΔPSI for RI in male and female flies (Figure 3B). Irrespective of the magnitude of change in PSI, all RI event except one showed an increase in PSI in TBI flies compared to non-treated control for male and female flies.

Gene ontology analysis for all genes (*N* = 374) containing significant differential RI events showed enrichment of genes mapping to neuronal processes such as synaptic vesicle transport and endocytosis (FDR ≤ 0.1) (Supplemental Figure 1). We anticipated intron retention would impact genes associated with synaptic processes. Manually curating genes containing RI revealed several interesting genes that may explain the reduced long-term survival in TBI flies. These genes include metabolic genes such as Isocitrate Dehydrogenase (Idh) (Figure 4A), Enolase (Eno), Pyruvate kinase (Pyk), and Aconitase (Acon), and synaptic transport proteins such as Stoned A (StnA) (Figure 3C).

**Figure 5:**
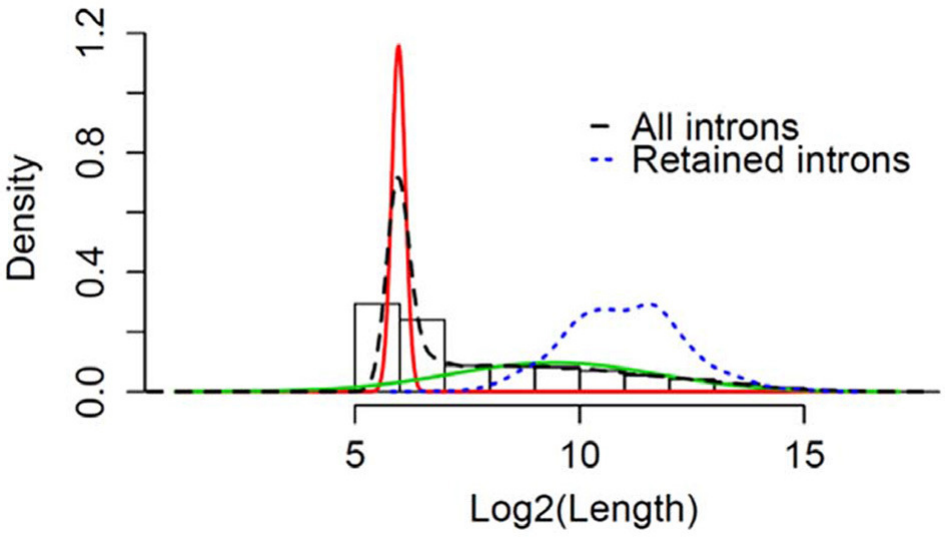
Retention of long intron post-TBI: Graphical representation of Gaussian mixture model to determine the natural cut-off for calling long introns. Black dotted line represents the log2(intron length) for all introns in the genome. Blue dotted line represents the intron length of retained introns detected for heads collected 24 h post-TBI for males and females. The red and green line represents the normal distributions fitted using Gaussian-mixture model (Pelosi et al., 2015).

We wanted to determine if the RIs observed commonly in male and female flies can be classified based on their length. Using a Gaussian mixture model the natural cut-off for long introns was determined to be >81 bps (Figure 5). All the RIs observed in male and female flies were classified as long introns, with a mean size of ~3,000 nucleotides (Blue dotted line, Figure 5).

We validated the RI events with qRT-PCR for three genes, Eno, Pyk, and StnA. Validation was done by using one primer in either exon 1 or exon 2 and one primer in the first intron which produces a 200-400 nucleotide amplification product. As expected, we found a significant increase in intron retention for all three of these genes by qRT-PCR (Supplemental Figure 5; Supplemental Table 1). We note that the fold-change we observed in RI, which ranged from 1.5-fold for Eno1, 2.5-fold for StnA, and 7.0-fold for Pyk, are probably underestimates, as unspliced mRNAs are usually subject to nonsense mediated decay and degraded (Chang et al., 2007; Isken and Maquat, 2007; Chapin et al., 2014).

### Profile of mitochondrial activity 24 h post-TBI

**Figure 6:**
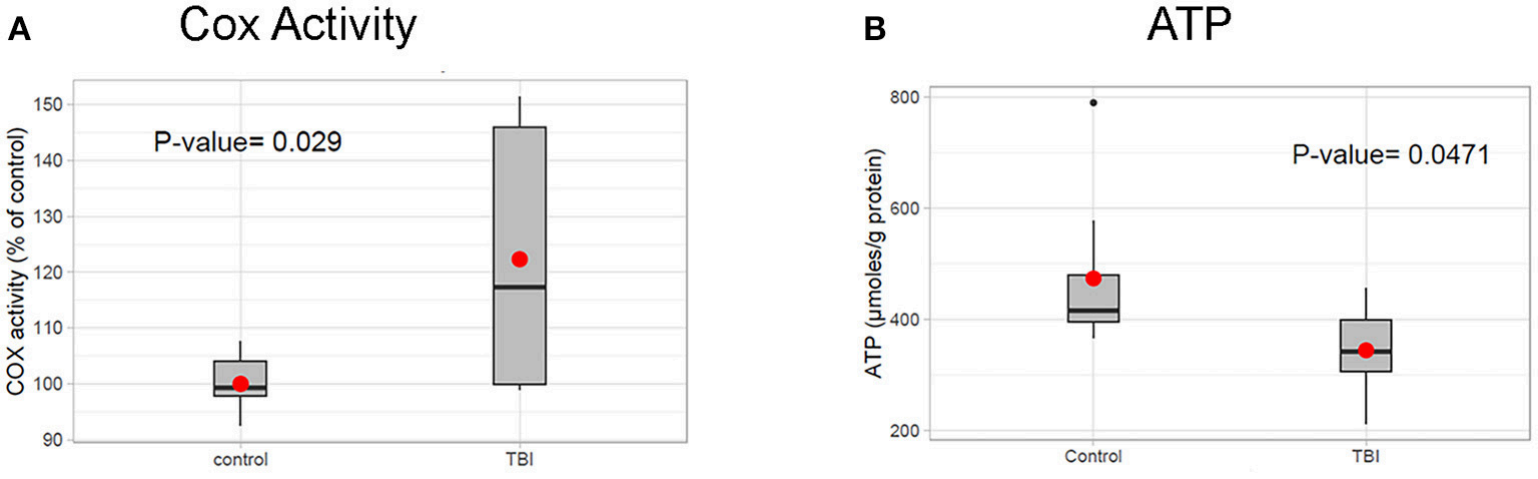
Mitochondrial profile post-TBI. **(A)** Effect of TBI on COX activity in total fly heads (see methods). COX specific activity of solubilized heads was measured using the polarographic method and is reported as % of control. The 24 h post-TBI heads show an 22% increase in COX activity (*p*-value (*t*-test) = 0.029). **(B)** ATP levels in fly heads were determined using the bioluminescent method and normalized to protein content. Heads collected 24 h post-TBI shows a significant decrease (*p*-value (*t*-test) = 0.0471) in total ATP concentration (μmol, ATP/mg Protein) compared to the control.

Based on the RI results in nuclear encoded mitochondrial metabolism genes such as Idh, Eno, and Pyk presented above, we hypothesize that mitochondrial altered is reduced by TBI. Additionally, TBI is known to cause an increase in mitochondrial stress and a corresponding increase in energy demand in damaged tissues for tissue remodeling. To determine whether mitochondrial activity is altered by TBI, we measured the activity of mitochondrial cytochrome c oxidase (COX), the terminal and rate-limiting enzyme of the electron transport chain 24 h after TBI. We found that COX activity increased by 22% in the TBI heads compared to controls (Figure 6A). This suggests that an increased energy demand is likely due to tissue remodeling after trauma, a process that is energy expensive. Analysis of ATP levels indicated a significant 27.2% decrease of cellular ATP in the 24 h post-TBI heads (Figure 6B). A drop of cellular ATP and concomitant increase in ADP would further activate COX since this enzyme is allosterically regulated by the ATP/ADP ratio (Arnold and Kadenbach, 1999). This result suggests a probable association between increased mitochondrial energy demand and RI in Kreb's cycle genes. This association needs to be further explored in future studies.

### Discovery of splicing factor binding motifs

Intron retention is presumably regulated by the interaction between splicing factors and splicing machinery. Introns contain intronic splicing suppressors or enhancers which bind RNA binding proteins and regulate their efficient processing and subsequent removal. We hypothesize that motif enrichment analyses of the RI introns would help us to identifying splicing factors that are involved in the regulation of RI by TBI. Therefore, we performed de-novo motif enrichment analysis using Discriminative Regular Expression Motif Elicitation (DREME), within 300 bps windows near the 5'SS and 3'SS of RIs.

DREME analyses revealed specific enrichment of CA-rich motifs RNA-binding motifs (Supplemental Figures 2, 3) (Bailey, 2011). This CA-rich RNA motif binds to Smooth (Sm), an hnRNP-L (heteronuclear ribonuclear protein-like) homolog, or Cnot4, an RBP protein of unknown function (Ray et al., 2013). We also found other motifs for RBPs such as Shep (AT-rich motif), Aret (GT-rich motif) and Rbp9 (T rich motif) near the 5'SS (Supplemental Figure 1), as well as Shep (AT-rich motif) and SF1 near the 3'SS (Supplemental Figure 2). Motif analyses were performed using reshuffled 300 sequences as background.

### Smooth may play a role in regulating intron retention post-TBI

We chose to focus on Smooth (Sm) in this study rather than the other RNA-binding proteins for several reasons: (1) the CA-rich motif is correlated with RI in a parallel study in our laboratory using a mouse spinal cord injury model (D.M.R., manuscript in preparation); (2) Sm-mutant and over-expressing stocks are available from the stock center; (3) the Sm-ortholog in humans, HNRNPL, associates with SET2 (Yuan et al., 2009), the H3K36 histone methyltransferase; and (4) H3K36me3 demarcates exons from introns, and regulates some forms of alternative splicing (Barrand et al., 2010; Kim et al., 2011; Zhao et al., 2017), which might explain the RI events that we observe after TBI.

To understand the possible role of Sm in regulation of splicing events we obtained the *sm*^4^ mutant stock. Briefly, the *sm*^4^ mutant has an abnormal phenotype over a deficiency (*Df*) (Karpen and Spradling, 1992). These *sm*^4^/*Df* flies survive to adulthood but are short lived with a median age of about ~30 days, and have synaptic defects in the neuromuscular junctions (Layalle et al., 2005). After confirming the short-lived phenotypic characteristic of the *sm*^4^/*Df* flies (data not shown), we subjected them to 1 strike on the HIT device, collected the fly heads 24 h after trauma, and performed RNA-seq. We estimated the ΔPSI (percent splicing in) using MISO compared to the non-treated controls separately for male and female flies and filtered them using ΔPSI ≥ ±5% and Bayes factor ≥10 (Katz et al., 2010). We only considered the events that overlapped between males and female flies for further analysis.

**Figure 7:**
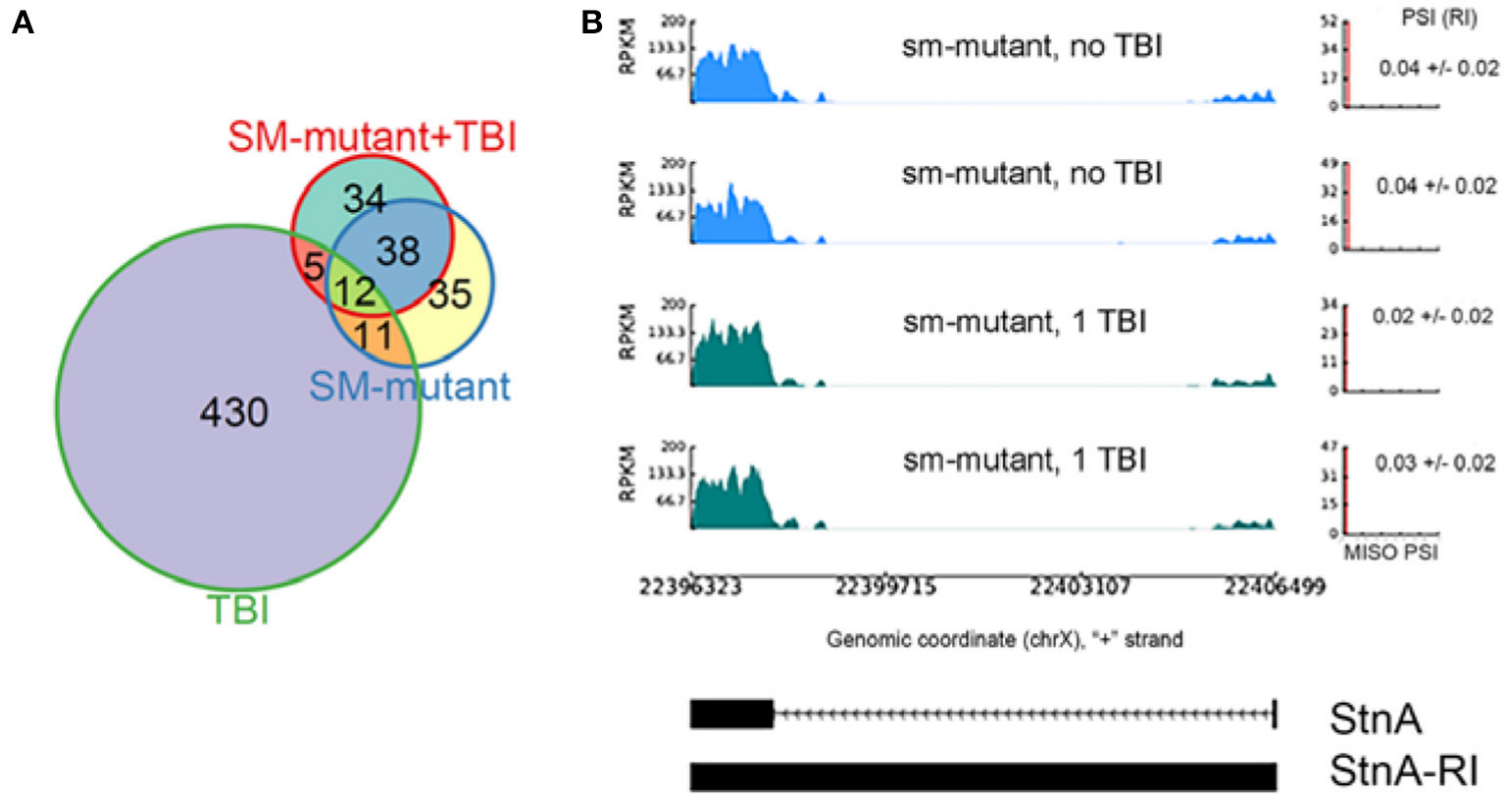
The *sm*^4^-mutation abolishes the intron retention events observed in TBI fly heads; **(A)** Overlap between differential RI events (ΔPSI) detected in TBI and *sm*-mutant+TBI samples. **(B)** RI events detected in TBI samples revert back to the non-TBI *w*^1118^ controls in SM-mutant and *sm*-mutant+TBI samples for Stoned A (StnA). Notice that even the background levels of RI are eliminated in the sm-mutant flies, and that the PSI levels do not increase after TBI.

We found 96 RI events detected both in male and female *sm*^4^/*Df* mutant flies. Roughly, 50% of these events were also detected in *sm*^4^/*Df* mutant flies inflicted with TBI (Figure 7A). Two of the most striking examples was observed in StnA (Figure 7B) and Idh (Figure 4), where *sm*^4^/*Df* mutation nearly completely abolished the RI. Notice that even the basal levels of RI in StnA and Idh are virtually eliminated in the *sm*^4^/*Df* mutant fly heads (Compare Figure 7B with Figure 3C).

A Smooth over-expression stock is also available from the Zurich ORFeome Project, which comprises about 2850 genes in a collection of 3xHA tagged ORF variants (Bischof et al., 2014). We planned to use this insertion to determine if we can rescue the *sm*^4^/*Df* mutant flies or alter RI when it is over expressed. Unfortunately, we could not conduct these experiments because larvae and adult flies were not viable when *UAS-sm.HAx3* flies were crossed to a large number of neuronal and pan-Gal4 drivers, such as *elav-Gal4* or *da-Gal4*, even at 18°C, which generally lowers Gal4 levels.

### Kdm4A, a H3K36me3 de-methylase, causes intron retention in a manner like TBI

Because H3K36me3 is known to be involved in regulating alternative splicing, we wanted to determine whether the demethylase that removes the methyl groups on H3K36me3 affects alternative splicing, and might be involved in promoting RI events after TBI. Also, the mitochondrial activity deficiency we observed, combined with the reduction in Idh and other mitochondrial metabolic genes by RI, lead us to test the attractive hypothesis that the activity of Jumanji-family of histone demethylases is reduced, because this family of proteins requires α-KG as a co-factor, which is made by the Idh gene (see Discussion).

Therefore, we investigated the effects of *Kdm4A* mutations on alternative splicing, because Kdm4A, a Jumanji-C (JmjC) domain containing histone demethylase, removes H3K36me3 from the Drosophila genome (Lin et al., 2012; Crona et al., 2013). Also, H3K36me3 has been associated with regulation of alternative splicing (Luco et al., 2010; Pradeepa et al., 2012; Zhou et al., 2014). Therefore, we hypothesize that knock-down of *Kdm4A* will cause significant changes in alternative splicing. Homozygous loss of function mutations of *Kdm4A* in Drosophila has been shown to cause developmental arrest (Tsurumi et al., 2013). Therefore, we attempted to knockdown *Kdm4A* in adult heads with *UAS-Kdm4a.RNAi* lines crossed to either *elav-Gal4, a pan-neuronal Gal4 driver*, or *da-Gal4*, a ubiquitous Gal4 driver, but the adult flies did not survive in either condition, and most of the larvae died at the 3rd instar larval stage. Previous studies have reported that Kdm4A is well expressed in 3rd instar larvae (Lorbeck et al., 2010). Therefore, to define the role of Kdm4A on splicing regulation irrespective of physical trauma, we decided to continue our study with 3rd instar larvae.

**Figure 8:**
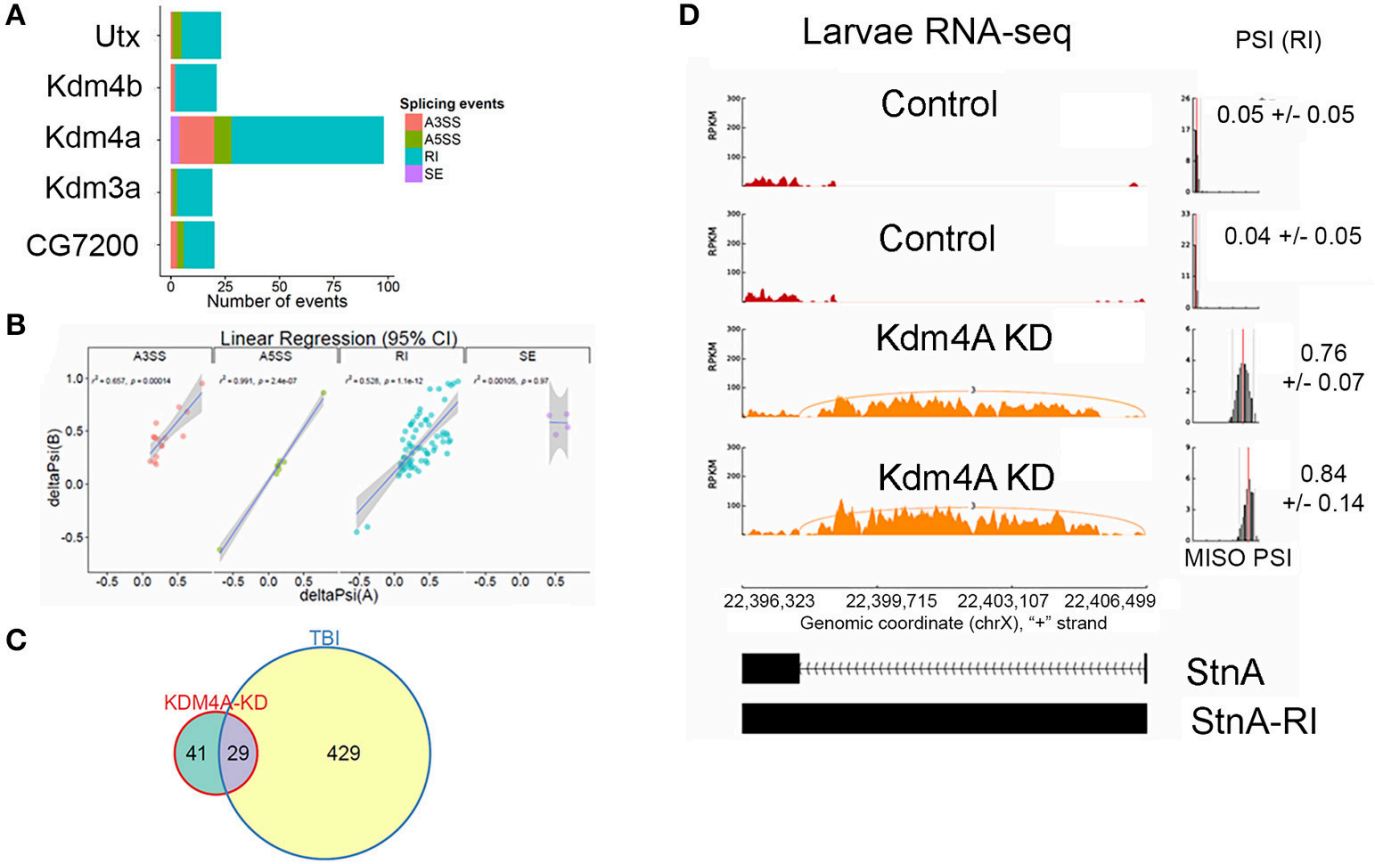
Knockdown of Kdm4A in 3rd instar larvae causes intron retention. **(A)** Effects of RNAi reduction of Jumanji family histone demethylases Ultratrithorax (dUtx), Kdm4b, Kdm4a, CG8165, and CG7200. Notice that Kdm4a, which is specific for H3K36me3, has the largest number of alternative splicing events. **(B)** Linear regression between ΔPSI or deltaPSI for differential splicing events detected for Kdm4A-mutant samples. **(C)** Overlaps between statistically significant differential alternative splicing events detected using MISO for heads collected 24 h post-TBI for males and females and dKDM4A-mutant in 3rd instar larvae. **(D)** sashimi plot showing intron retention event in StnA. The data is represented as Reads Per Kilo-base per Million (RPKM). Notice that the PSI values increase dramatically in the Kdm4A KD larvae (*p* < 0.0001).

We knocked down *Kdm4A* in 3rd instar larvae using the UAS/GAL4 RNAi system, with the *da-Gal4* ubiquitous driver, where we achieved over 90% reduction in mRNA levels, (Supplemental Figure 4), and performed RNA-sequencing using 100 bps paired-end reads. Kdm4A activity has been reported to be associated with transcriptional repression in mammalian cells (Zhang et al., 2005; Huang and Dixit, 2011). Comparing the alternative splicing profile of *Kdm4A-RNAi* against the control (*w*^1118^) 3rd instar larvae we found 98 alternative splicing events, which were well-correlated between biological replicates (Figure 8A).

Analysis of UAS-RNAi knock downs of other Jumanji-histone-demethylase family members, *Ultratrithorax* (*Utx*), a H3K27me3 demethylase (Smith et al., 2008; Copur and Müller, 2013), *Kdm4b*, a H3K9me3 demethylase (Tsurumi et al., 2013), *Kdm3a*, a H3K9me3 demethylase (Herz et al., 2014), and *CG7200*, a Jumonji-family member with unknown histone-demethylase specificity, in 3rd instar larvae with *da-Gal4* did not show nearly as many alternative-splicing events as knocking down *Kdm4a* (>5x fewer; Figure 8A), suggesting that H3K36me3 is one of the most important H3 modifications in regulating alternative splicing.

The alternative splicing events in *Kdm4a* knock-down larvae showed a significant change in ΔPSI values (ΔPSI ≥ |0.05| or |5%| and Bayes Factor ≥ 10). Among most of the events (70/98) were RIs (Figure 8B). These events showed a significant ~52% positive correlation between replicates, and an overlap with RI events seen in TBI (Figure 8C). Notably, 29/70 events were also detected in 24hrs post-TBI, including RIs detected in metabolic regulators such as Isocitrate dehydrogenase (Idh) (Figure 4) and Pyruvate Kinase (Pyk), in addition to genes responsible for synaptic vehicle endocytosis such as Stoned A (StnA) (Figure 8D). This provided us with initial evidence that H3K36me3 might play an integral role in regulating RIs, possibly by associating with Smooth at CA-rich repeats in the introns of long RNAs.

### H3K36me3 is associated with splicing changes in the 24 h post-TBI

**Figure 9:**
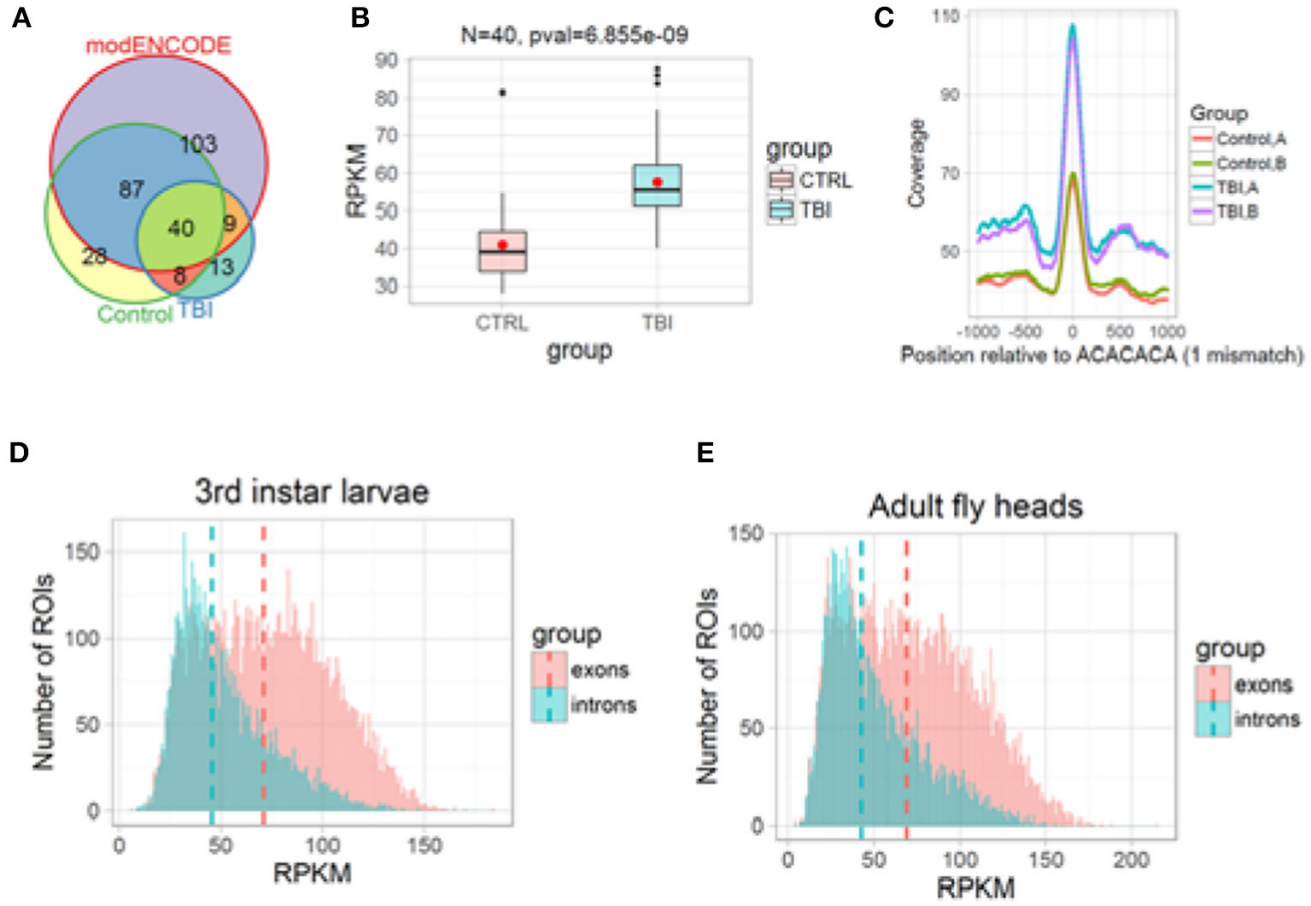
H3K36me3 increases in Smooth binding sites in large introns after TBI. **(A)** H3K36me3 ChIP-seq peaks for Control, TBI, and modENCODE H3K36me3-ChIP-seq data were overlapped. We found 40 RI in common. **(B)** For these 40, average RPKM for all H3K36me3 ChIP-seq peaks were estimated for Control and TBI samples. These 40 intron showed an significant increase in H3K36me3 in TBI samples (*p* = 6.85e-09, Welch 2-sample *t*-test). **(C)** We remapped our significant peaks and centered it around CA-rich motifs (ACACACA with 1 mismatch). The bar at the bottom of each track shows the location of the CA-centered significant peaks. Notice that TBI significantly increases the amount of H3K36me3 at ACACACA sequences (*p* < 0.001, Welch 2-sample *t*-test). **(D,E)**, Histogram of mean normalized counts from ModENCODE chip-seq data on introns and exons for 3rd instar larvae **(D)** and Adult fly heads **(E)**. The differential tag densities within 200 bp regions was estimated using MEDIPS. Regions which showed a ≥ + 2-fold enrichment at an FDR corrected *p* ≤ 0.05 over input, was considered as significant peaks. The target peaks were overlapped with regions of interest (ROIs). Criteria for selecting ROIs are as follows; introns and exons containing significant peaks was ≥200 bps in length. Reads Per Kilobase per Million (RPKM) for respective ROIs were plotted in R/Bioconductor.

Knocking down *Kdm4a*, which should increase H3K36me3 levels, increases intron retention in many of the same genes as TBI (Figure 8C), suggesting that TBI might also increase RI by increasing H3K36me3 levels in introns. To determine the characteristic profile of H3K36me3 in Drosophila tissues, we used archived H3K36me3 chromatin-immunoprecipitation followed by next-generation DNA-sequencing (ChIP-seq) (35 bps single-end) data for 3rd instar larvae (L3) (GSE47248) and heads (GSE47280) from the modENCODE consortium. We called significantly-enriched peaks against the respective input controls within 200 bps non-overlapping sliding windows across the entire genome. Then we filtered them using a FDR corrected *p* ≥ 0.05 and log_2_FC ≥2. Finally, we calculated the mean RPKM of significant peaks across disjointed and unique exonic and intronic regions of transcripts and plotted their density distributions using R/Bioconductor. Our analysis indicated that exons are generally marked by a higher level of H3K36me3 compared to introns in Drosophila 3rd instar larvae and adult heads (Figures 9D,E).

Based on our reanalysis of modENCODE ChIP-Seq data and KDM4A knockdown studies in 3rd instar larvae, we hypothesized that post-TBI there is an increase in H3K36me3 within the introns in the RI genes in the heads. To confirm this hypothesis, we performed ChIP-seq for H3K36me3 in *w*^1118^ control and TBI heads and sequenced them using 100 bps paired-end reads. The long 100 bps paired-end reads are advantageous as they allow better estimation of the immunoprecipitated fragment size distribution than the 36 bps reads in the modENCODE dataset (~176 bps for all samples). We called enriched peaks against input controls within 200 bps sliding windows using an FDR corrected *p*-value cut-off of 0.1 (10%) for *w*^1118^ control and 24 h post-TBI fly heads (see Methods).

Following peak annotation, we calculated the mean-RPKM of H3K36me3 peaks within our RIs (see Methods). The RIs which contained a minimum of 8 reads per samples were considered as represented in our final dataset. We detected 48/458 RIs which contained significant peaks in the control (*w*^1118^) and 24 h post-TBI heads (Figure 9A). To provide an independent validation of our H3K36me3 ChIP, we also calculated the mean-RPKM of significant peaks for modENCODE H3K36me3 heads (GSE47280) within RIs. Again, only RIs with minimum of 8 reads per sample were selected for further study. 40/458 RIs were represented in control (*w*^1118^), 24 h post-TBI heads and modENCODE H3K36me3 ChIP-seq datasets (Figure 9A). For these 40 RIs, the mean-RPKM of H3K36me3 peaks for 24 h post-TBI heads were significantly higher compared to the control (*w*^1118^) heads as illustrated in Figure 9B.

To understand the relationship between Sm and H3K36me3 we centered our H3K36me3 intronic peaks on the CA-rich motif (ACACACA) (see Methods). The ACACACA motif positions (100 bps centered regions) surrounded by significant H3K36me3 peaks (±1,000 bps) for control and 24 h post-TBI heads are indicated in Figure 9C. We observed that the heads collected 24 h post-TBI showed higher levels of H3K36me3 in the RIs compared to the controls. Furthermore, ACACACA motif position have notably higher H3K36me3 compared to surrounding regions in both Control and 24 h post-TBI samples. This observation suggested a possible interaction between H3K36me3 and Sm-binding.

## Discussion

In the Drosophila TBI model, we observed RI in genes associated with the TCA cycle such as Idh and Acon and neuronal transport genes such as StnA. Previous studies have demonstrated that RI targets transcripts to nonsense-mediated decay (NMD) through introduction of a pre-mature termination codon (Chang et al., 2007; Isken and Maquat, 2007). We also observed a significant upregulation of COX (cytochrome C oxidase) activity 24 h post-TBI in pooled male and female fly heads which suggests a loss of ATP-dependent inhibition (Kadenbach et al., 2004). COX is the last enzyme of the respiratory electron transport chain and is responsible for conversion of molecular oxygen to water. During this process, the pumps transport protons across the inner mitochondrial membrane, generating an electrochemical gradient, which is utilized by ATP synthase to produce ATP. Under normal conditions, COX activity is controlled by allosteric inhibition by ATP (Arnold and Kadenbach, 1997). Under oxidative stress conditions, the allosteric inhibition is lost, resulting in hyperpolarization of the membrane potential and production of ROS.

Loss of COX inhibition results in the inability of a cell to sense ATP and therefor ATP is not modulated per demand. This phenomenon has been noted in neurodegenerative conditions and is classified as a known hallmark of oxidative stress (Arnold, 2012). We also observed a decrease in ATP availability 24 h after TBI. This observation is counter-intuitive because an increase in COX activity is expected to generate more ATP. Repair and tissue remodeling after TBI is an energy expensive process and it is expected to increase utilization of available ATP. Our preliminary investigation seems to indicate that the ATP expenditure due to tissue repair might be disproportionately more expensive than increased ATP production due to loss of COX inhibition. Disruption of mitochondrial homeostasis and dysregulation of TCA cycle genes might culminate in a high stress environment for neurons and may contribute to development of progressive neurodegeneration in the Drosophila model of TBI.

Acon and Idh are critical epicenters that regulate neuroprotection in neurodegenerative conditions. Dysregulation of steady state RNA levels of these genes can significantly impact the production of a critical metabolite that is involved in epigenetic regulation, such as α-Ketoglutarate (α-KG) (Chia et al., 2011). α-KG is an important co-factor in regulating all dioxygenase reactions in the cell, including Kdm4A, the H3K36me3 demethylase. Direct perturbation of α-KG/succinate levels is sufficient to cause changes in H3K27me3 and DNA methylation in mouse embryonic stem cells, suggesting α-KG is an important metabolic effector of epigenetic modification by both histone and DNA demethylases (Carey et al., 2015). We speculate that downstream impact of dysregulation of TCA cycle genes might be mediated through modulation of α-KG levels.

Finally, in this study we present preliminary evidence for the role of hnRNP-L/Sm in regulation of RI. The hnRNP-L splicing factors are versatile in their function and can regulate a wide variety of splicing events, including skipping of cassette type exons and suppression of variable exons. Notably, hnRNP-L knockdown and hnRNP-L/hnRNP-LL double knockdown causes RI in CD55 and STRA6 genes in HeLa cell lines, suggesting that they may be required for efficient processing of introns (Hung et al., 2008). The Drosophila genome encodes a single homolog of the hnRNP-L protein, known as Smooth (Sm). Sm contains 3 protein motifs corresponding to RRM (RNA binding) domains which share 42, 25, and 42% identity with RRM domains of human hnRNP-L proteins. *Sm*^4^-mutants have an average lifespan of 30 days, compared with 60–90 days for wild type flies, due to defective feeding behavior (Layalle et al., 2005; Draper et al., 2009) that has been attributed to the lack of chemosensory axon arborization in the leg neuromeres (Layalle et al., 2005; Draper et al., 2009). Reduced fitness and loss of RI in *sm*-mutant flies independent of TBI, suggest that RI might be critical for neuroprotection and might modulate steady state RNA levels of critical neural and metabolic genes.

### Model for intron retention induction by TBI

**Figure 10:**
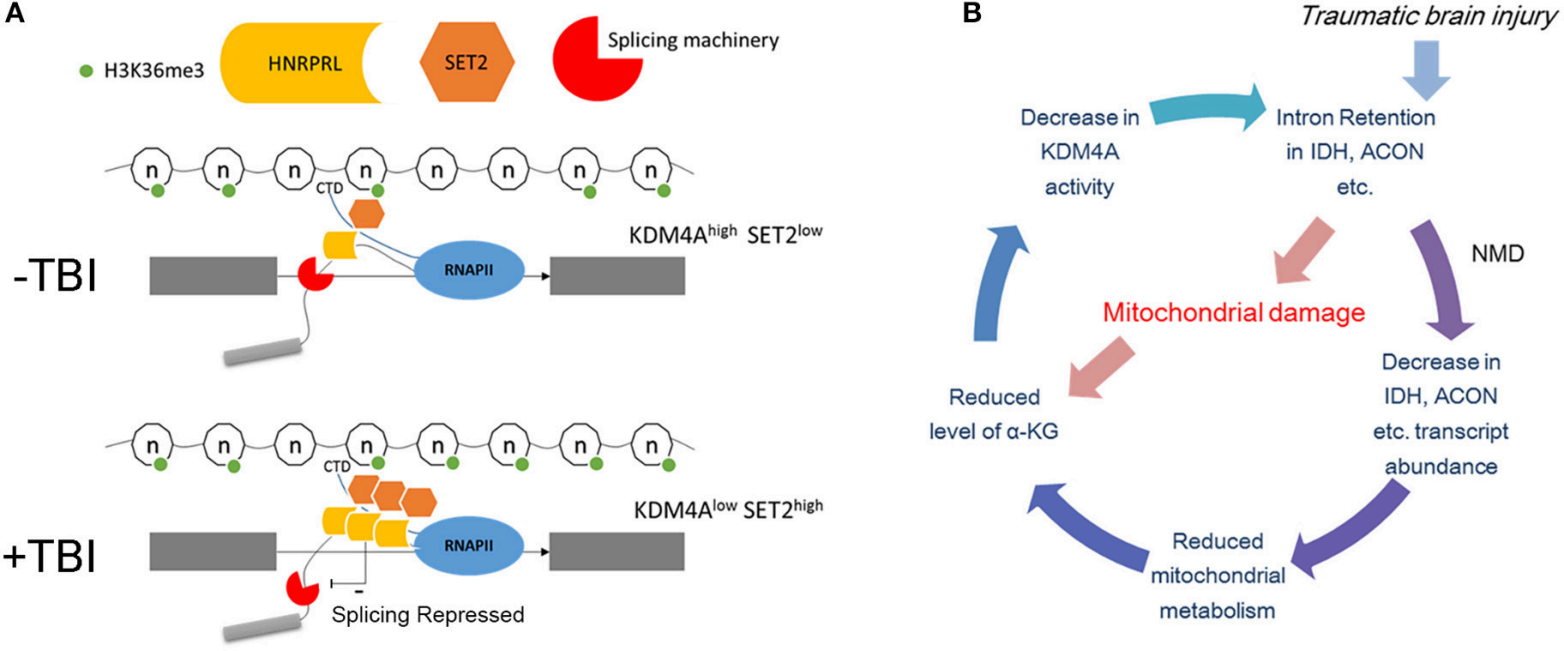
Possible mechanism of intron retention. **(A)** Top panel: Under normal conditions the introns are marked by lower levels of H3K36me3 compared to the flanking exons. This allows the elongating polymerase and the associated splicing machinery to recognize it as an intron. The binding of hnRNP-L/Smooth to intronic splicing suppressor sites under normal conditions is limited and cause only a basal level of intron retention. Bottom panel: Post-TBI the level of H3K36me3 in introns increases and now it resembles an exon therefore is spliced in by the splicing machinery. This may result in recruitment of hnRNP-L/Smooth to intronic splicing suppressor sites. **(B)** Possible negative feedback loop for increasing H3K36me3 levels. In this model, TBI damages the mitochondria which reduces the levels of α-KG. This leads to lower Kdm4A activity, which increases the levels of H3K36me3 in the long intron of Idh, Acon, and other metabolism genes. Nonsense-mediated decay (NMD) decreases the amount of unprocessed Idh mRNA, which further reduces α-KG levels.

We attempt to integrate our findings in this paper in a model for how TBI causes intron retention in Drosophila (Figure 10A). First, we showed that mitochondrial activity decreases, and we speculate that this might lead to localized reduction in α-KG levels in certain neurons. Next, we found that mutations in either *sm*, the hnRNP-L ortholog, eliminates RI in the long introns with CA-repeats that are retained after TBI. In contrast, we found that RNAi knock down of *Kdm4a*, which demethylates H3K36me3, increases RI in many of the same introns by increasing H3K36me3 levels in these introns. In our model, we speculate that a reduction in α-KG levels after TBI, caused by the observed decreased mitochondrial activity, combined with RI in the Idh and Acon genes, could lead to a decrease in Kdm4A activity, which requires α-KG as a cofactor. Kdm4A, as well as other dioxygenases in the Jumonji family, requires α-KG as a co-factor, which is produced by the Idh gene in the mitochondria. The loss of Kdm4A activity, we propose, increases H3K36me3 levels in introns, thereby, making them resemble exons by the mRNA splicing machinery (Figure 10B). Additionally, the increased H3K36me3 levels in the long introns may result in recruitment of hnRNP-L/Smooth to intronic splicing suppressor sites, and thereby cause the introns to be retained.

### Future studies

The evidence presented in this paper raise several interesting unanswered questions which need further investigation: (1) How long does it take for RI to reduce steady state RNA levels, (2) How is RI associated with mitochondrial metabolism, (3) What provides the initial signal for Sm to bind to long introns post-trauma, and (4) is the RI phenotype a protective mechanism that reduces the severity of TBI? In future studies, we will continue to explore these questions and further our understanding of regulation of alternative splicing in the sub-acute phase of TBI, and to determine whether this mechanism is preserved in mammals.

## Methods

### Drosophila stocks and traumatic brain injury methods

Sample consisted of 0–5 days old *w*^1118^ flies. These flies were collected in sturdy plastic vials and subjected to TBI using the high impact trauma (HIT) device. For the survival assay, the male and female flies were separated and 25 flies per vial were used for each condition (control, 1 strike, 2 strike). The flies were subjected to TBI, at a spring deflection of approx. 45° to ameliorate the impact of trauma. The 2nd strike was performed after a recovery time of 5 mins. These flies were maintained till ~98% of the flies in the control vial were deceased. Survival estimations and standard error calculation was done using R/Bioconductor. For RNA sequencing analysis heads from 50 males and 50 females flies for control, 1 strike and 2 strikes were pulled off manually one at a time and immediately transferred to RNAlater (500 μl). Fly head collection was done at 24 h after TBI. The smooth-RNAi lines were not viable when crossed to da-Gal4, so instead we used a hypomorphic allele over a deficiency in a hemizygous combination; cn[1] P[PZ] sm[05338]/CyO; ry[506] (aka. sm[4]) and w[1118]; Df(2R)BSC820, P+PBac w[+mC] = XP3.WH3 BSC820/SM6a. The *sm*-mutant flies were subjected to a single strike and heads were collected 24 h post trauma.

Most of the gene knockdowns were performed by crossing da-Gal4 flies, which have ubiquitous expression, to UAS-RNAi flies using the following stocks from the Bloomington, Indiana stock center: y[1] sc[^*^] v[1]; Py[+t7.7] v[+t1.8] = TRiP.HMS01304 attP2 (expresses dsRNA for RNAi of Kdm4A (FBgn0033233) under UAS control); y[1] v[1]; Py[+t7.7] v[+t1.8] = TRiP.HMS02273 attP40 (expresses dsRNA for RNAi of Idh (FBgn0001248) under UAS control); P(GAL4-da.G32), which expresses Gal4 in all cells; y[1] v[1]; Py[+t7.7] v[+t1.8] = TRiP.JF01320 attP2 (Expresses dsRNA for RNAi of Kdm2 (FBgn0037659) under UAS control); y[1] sc[^*^] v[1]; Py[+t7.7] v[+t1.8] = TRiP.HMS00488 attP2 (Expresses dsRNA for RNAi of CG7200 (FBgn0032671) under UAS control); y[1] sc[^*^] v[1]; Py[+t7.7] v[+t1.8] = TRiP.HMS00775 attP2 (Expresses dsRNA for RNAi of CG8165 (FBgn0037703) under UAS control); and y[1] sc[^*^] v[1]; Py[+t7.7] v[+t1.8] = TRiP.HMS00575 attP2 (Expresses dsRNA for RNAi of Utx (FBgn0260749) under UAS control).

### Tissue homogenization

For tissue homogenization 4 stainless steel 3.2 mm beads (Qiagen cat no. 69990) were added to 2 ml Eppendorf tubes. The samples were subjected to homogenization using TissuelyserLT (Qiagen) using the following setting; 50 oscillations (1/s) for 2 min. The homogenized samples were centrifuged using a low-speed bench top centrifuge and supernatant containing cells were collected for downstream processing.

### RNA isolation

RNA was isolated from homogenized cells as described in Qiagen EZ1 RNA Handbook, 3rd ed. 06/2012, page 34, for “Any Type of Tissue” using the EZ1 RNA Universal Tissue Kit with TissueLyser II homogenization/disruption and optional DNase digestion. RNA was quantified by UV Spec on the Trinean DropSense96 Spectrophotometer. RNA quality assessment was performed using the Agilent RNA ScreenTape on the Agilent 2200 TapeStation.

### RNA-sequencing alignments

Prior to alignment the sequencing adaptors were trimmed using FASTQ Toolkit v1.0 on Illumina's BaseSpace portal. FastQC was used to generate quality metrics for assessment of fastq files (Andrews, 2010). RNA sequencing alignment was done using TopHat v2.1.0 or HISAT 0.1.6-beta using default alignment parameters (Trapnell et al., 2009; Kim et al., 2013). After alignment, the mapped reads was further filtered by mappability score (MAPQ ≥ 10). The quality controlled bam files were sorted by genomic position of the reads using samtools-0.1.19 (Li et al., 2009). PCR duplicate reads were processed and removed using rmdup function (options -S) in samtools. The sorted duplicate removed bam files were further assessed and visualized using Integrative Genome Viewer (http://software.broadinstitute.org/software/igv/). For Drosophila Melanogaster UCSC genomic build dm3 was used for read alignments.

Reads from the processed bam files were overlapped with Ensembl exon annotation extracted from UCSC in R/Bioconductor (Packages; GenomicAlignments, GenomicFeatures). The dm3 genomic-build was used for Drosophila. The read count per exon was computed using the preset “Union” mode. The exon counts were next combined to give the gene read counts. Differential gene expression analysis from computed read counts was carried out using DESeq2 (Love et al., 2014).

For Drosophila model of TBI RNA were prepared in bulk for 50 males and 50 females for 2 separate conditions; control and 1 strike at 24 h. The differential expression analysis was performed in between TBI flies and non-TBI controls using sex as a covariate (read distribution ~ conditions + sex) (Love et al., 2014). Differentially expressed genes were further filtered using FDR ≤ 0.1 and log2 FC ≥2.

### Alternative splicing analysis

We used Mixture of isoform (MISO) to estimate the Percent Spliced in (PSI) value for each annotated splicing feature (Katz et al., 2010). Annotations for splicing features were provided by modENCODE consortium and classified into 5 representative classes; alternative 3'SS, alternative 5'SS, skipped exons, mutually exclusive exons, and retained introns. The difference in PSI value (ΔPSI) between control and treatment (TBI) samples were estimated using Bayesian factor analysis. The comparisons were further filtered using a ΔPSI cut-off of 0.05 or 5%, Bayesian factor ≥10 and number of exclusion and inclusion reads ≥10 (see MISO documentation). The significant events common between males and females were selected and correlated to give the final list of sex-independent splicing changes. As majority of the significant events were retained introns downstream annotation and visualization was done only with retained intron genes. The retained introns were overlapped with maximum overhang length of 10 bps with introns annotation obtained from the ENSEMBL genes UCSC dm3 build of the genome and annotated with respective gene and transcript annotation. For visualization gene specific alternative splicing events log10 (RPKM) (reads per kilobase per million) for respective splicing features were plotted using sashimi plot (https://miso.readthedocs.io/en/fastmiso/).

### Characterization of introns

Introns and their respective lengths in base-pairs were obtained for ENSEMBL genes from UCSC dm3 build of the Drosophila genome. The lengths were log transformed (log2) and their density distribution was determined. Then the density distribution was modeled as a mixture of *N* = 2 normal distribution using Gaussian mixture model. This allowed us to determine the natural cut-off for long introns. The distributions inferred from the model were plotted using R/Bioconductor.

### Splicing factor discovery

We limited our motif discovery to intronic splicing sites, i.e., motifs within RIs. The median intron size of retained introns was ~3,000 bps (min = 115, max = 36,560). Therefore, only retained introns with size ≥600 bps (421/458) was considered for motif analysis. For potential splicing factor binding motifs, 300 bp regions were collected from the intronic side of the 5'SS and 3'SS. Sequences for these target regions were obtained and Motif enrichment analysis was carried out using DREME (Discriminative Regular Expression Motif Elicitation) (Bailey, 2011). DREME uses reshuffled input sequences as control to calculate motif enrichment.

### Chromatin immunoprecipitation

The homogenized tissue collected was fixed by incubating with 16% Formaldehyde (final concentration 1%) for 10 min at RT. Then 10XGlycine (final concentration 1X) was added and samples were incubated at RT for 5 mins, to quench the activity of Formaldehyde. After incubation the samples were centrifuged at 3,000 g for 5 min and supernatant was removed to obtain cell pellets. The formaldehyde fixed cell pellets were washed 2 times with PBS (1X)+ HC (3,000 g for 5 min) and then reconstituted in Membrane extraction buffer + HC (Pierce Chromatin Prep module, 26158). Cell pellets were broken up by pipetting with a p200 and vortexing for 15 s and incubated on ice for 15 min. The lysed cells were recovered by centrifugation (9,000 g for 3 min at 4°C) and reconstituted in 200 or 300 μl of Nuclease free water (depending upon the size of the pellet, decided arbitrarily). Sonication was carried out using Covaris S2 Series 2 Focused Ultra-sonicator at intensity = 5, duty cycle: 10%, 200 cycle/burst, for 5-8 mins (depending upon size of the pellet and presence of debris). The sonicated nuclei were recovered using centrifugation (10,000 g for 5 min) and reconstituted in Nuclear extraction buffer + HC (Pierce Chromatin Prep module, 26158). The nuclei were incubated on ice for 15 min which 15 s of vortexing every 5 min. The nuclei were centrifuged at 9,000 g for 5 min and supernatant containing cleaved chromatin was collected for downstream analyses. The DNA concentrations were measured using Qbit. Chromatin bound DNA was collected from mass preps (*N* = 4) of control and TBI heads (24 h post-TBI) and pooled and redistributed for immunoprecipitation using Anti-Histone H3 (tri methyl K36) antibody - ChIP Grade (ab9050). We used total of 0.5 μg/reaction of DNA for control and TBI heads. The efficiency of the sonication and quality of IP was assessed using 2200 TapeStation System. The IP'ed DNA was sequenced using 100 bps paired-end reads in replicates. Quality control metric from sequencing runs were estimated using HOMER (http://homer.salk.edu/homer).

### Sample preparation for mitochondrial profiling

Zero to five days old w^1118^ flies were transferred to 50 ml tubes on ice. Approximately 3 ml of flies was collected per tube. These flies were snap frozen by dipping them in liquid nitrogen and vortexed vigorously to detach the head (for 3 min; 15 s per turn). The vial was dipped in liquid nitrogen between consecutive turns to prevent them from thawing. The vortex flies were passed through a sieve 720 μm pore size. This allowed the separation of the bodies from the detached heads. The detached heads were collected on another sieve 410 μm pore size. This sieve allowed the separation of heads from other detached body parts. The fly heads were transferred to 2 ml Eppendorf tubes kept on dry ice. Then they the stored at −80°C until measurement. We had a total of 16 control samples (0.038 ± 0.013 gm/tube) and 15 24-h post-TBI samples (0.018 ± 0.004 gm/tube).

### Cytochrome *c* oxidase (COX) activity measurements

To test the effect of TBI on electron transport chain function, COX activity measurements were performed as described (Lee et al., 2005, 2009). Briefly, fly heads were collected and stored frozen at −80°C until measurements were performed. COX activity was measured with a micro Clark-type oxygen electrode in a closed chamber (Oxygraph system, Hansatech) at 25°C. Frozen samples were minced in 10 mM K-HEPES, 40 mM KCL, 1% Tween-20, 2 mM EGTA, 1 mM Na-vanadate, 1 mM PMSF, 1 μM oligomycin) and solubilized by sonication for 5 s for 2 times. The supernatant was collected, and COX activity measured in the presence 20 μM cow heart cytochrome c (Sigma) and 20 mM ascorbic acid as reductant. Oxygen consumption was recorded on a computer and analyzed with the Oxygraph software. Protein concentration was determined with the DC protein assay kit (Bio-Rad, Hercules, CA, USA). COX specific activity was defined as consumed O_2_ (nmol)/min/mg total protein and reported as % of control.

### Measurement of total ATP concentration

ATP levels were determined via the bioluminescent Method (HS II kit, Roche Applied Science) in conjunction with the boiling Method as described (Samavati et al., 2008).

### RNA sequencing and chip sequencing

Library for RNA sequencing was prepared as described in the TruSeq Sranded Total RNA Sample Preparation Guide, Rev. E, Oct. 2013, using the TruSeq Stranded Total RNA LT (w/RiboZero Human/Mouse/Rat) -Set A library kit. 100 bps paired-end libraries for RNA and ChIP sequencing were sequencing on the Illumina HiSeq 2500 following cBot clustering.

### Chip-sequencing alignments

All ChIP sequencing alignments were done using *Bowtie2* using the dm3 build of the Drosophila genome as reference. After alignment, the mapped reads were further filtered by mappability score (MAPQ ≥ 10). The quality controlled. bam files were sorted by genomic position of the reads using *samtools*. PCR duplicate reads were processed and removed using rmdup function (options -S) in *samtools*. The sorted duplicate removed.bam files were further assessed and visualized using Integrative Genome Viewer (http://software.broadinstitute.org/software/igv/). The tag density and Quality metrics such as GC content and genomic nucleotide frequencies relative (−50, 50) to the 5′ end of ChIP-fragment were calculated using HOMER (http://homer.salk.edu/homer).

### Alignment and gene expression

Reads from the processed bam files were overlapped with ENSEMBL exon annotation extracted from UCSC in R/Bioconductor (Packages; *GenomicAlignments, GenomicFeatures*). The dm3 genomic-build was used for Drosophila datasets and mm10 genomic-build was used for the Mouse dataset. The read count per exon was computed using the preset “Union” mode. (Refer to *summarizeoverlaps*). The exon counts were next combined to give the gene read counts. Differential gene expression analysis from computed read counts was carried out using DESeq2 in R/Bioconductor. The read count and DESeq2 parameters used were identical between the Drosophila and mouse dataset.

For Drosophila model of TBI RNA were prepared in bulk for 50 males and 50 females for 3 separate conditions; control, 1 strike and 2 strikes at 2 time points; 4 and 24 h. This meant that for the exploratory analysis we had a total of 12 samples. The negative binomial distribution (see DESeq2 vignette) (Love et al., 2014) was fitted using treatment conditions; control, 1 strike and 2 strikes as classifiers and sex of the flies and time of collection as categorical covariates (read distribution ~ conditions + sex + time of collection). This enabled us to control for any potential sources of gene expression variations which may arise due to the sex of the flies. Additionally, this allowed us to use the male and the female w^1118^ flies were used as biological replicates. Male and female w^1118^ non-TBI flies collected at 4 and 24 h were used as controls for all differential expression estimations. The differentially expressed genes were further filtered using FDR and log2FC cut-off which are further discussed in the Results section.

### Exon usage analysis

Number of aligned reads was counted within disjoint exonic bins rather than exons using the “Union” mode of read-counting in *summarizeoverlaps*. Then DEXSeq was used to determine relative exon-usage while controlling for overall gene expression. Relative exon usage can be defined as follows;

Exon usage = # transcripts from the gene that contain target exon/# all transcripts from the gene.

For Drosophila TBI the negative binomial distribution for modeling read counts (see DEXSeq vignette) was fitted using treatment conditions; control, 1 strike and 2 strikes as classifiers and sex of the flies and time of collection as categorical covariates. For each target gene DEXSeq models read distribution as a function of ~conditions + exon + sex:exon+ time:exon + conditions:exon, where “:” indicate existence of possible interaction/correlations between covariates. Then this complete model is compared against a *null* model; ~conditions + exon + sex:exon+ time:exon, to determine the effect of treatment conditions of exon usage. Further details on DEXSeq can be found in the package vignette and the paper (Anders et al., 2012).

### Quantitative real-time PCR (qRT-PCR) validation of RI in three genes

We validated RI increases after TBI in 3 genes, Eno, Pyk, and StnA, by qRT-PCR. We used one oligo in either exon 1 or exon 2 and one oligo in intron 1 to PCR-amplify cDNA isolated from control brains or brains harvested 24 h after TBI. The results are shown in Supplemental Figure 5 and Supplemental Table 1. Thermo Scientific Verso 1-step RT-qPCR Low ROX Kit for SYBR Green was used to synthesize cDNA and perform the qPCR in one reaction. A master mix was made using the following volumes: 0.1 μL Verso Enzyme Mix; 5 μL 2x 1-step qPCR SYBR Low ROX mix; 0.5 μL RT Enhancer; 0.1 μL Forward Primer (10 μM); 0.1 μL Reverse Primer (10 μM); 3.2 μL nuclease free water. 1 uL of RNA template was added to 9 uL of master mix for a final concentration of 1 ng total RNA. The reactions were then run on the Applied Biosystems Quantstudio 12K Flex under the following cycling conditions: 50°C for 15 min; 95°C for 15 min; 40 cycles of: 95°C for 15 s, 57°C for 30 s and 72°C for 30 s.

## Author contributions

DR is the PI and supervised all aspects of this project. AS conducted most of the experiments and analyzed the data. KG helped with the data analysis. JL helped with the mitochondria experiments WQ helped with the splicing bioinformatics ON helped with the head collection and the bioinformatics RB helped with the head collection and the bioinformatics MH helped with the mitochondria activity RP-R helped supervise the bioinformatics.

### Conflict of interest statement

The authors declare that the research was conducted in the absence of any commercial or financial relationships that could be construed as a potential conflict of interest.
