# Supplementary material for: Smooth, an hnRNP-L Homolog, Might Decrease Mitochondrial Metabolism by Post-Transcriptional Regulation of Isocitrate Dehydrogenase (Idh) and Other Metabolic Genes in the Sub-Acute Phase of Traumatic Brain Injury
Source: Front Genet. 2017 Nov 15;8:175. doi: 10.3389/fgene.2017.00175 (PMC5694756; doi:10.3389/fgene.2017.00175)

SUPPLEMENTARY MATERIAL

Smooth, an HNRNPL homologue, might decrease mitochondrial metabolism by post-transcriptional regulation of Idh and other metabolic genes in the sub-acute phase of traumatic brain injury

Arko Sen[1,2], Katherine Gurdziel[3], Jenny Liu[4], Wen Qu[2], Oluwademi Okikiolu Nuga[2], Rayanne Burl[3,4], Maik Hutteman[4], Roger Pique-Regi[4,5], and Douglas. M. Ruden[1,3,5,6].

1, Institute of Environmental Health Sciences, Wayne State University, Detroit, MI 48201

2, Department of Pharmacology, Wayne State University, Detroit, MI 48201

3, C. S. Mott Center for Human Growth and Development, Department of Obstetrics and Gynecology, Wayne State University, Detroit, MI 48201

4, Center for Molecular Medicine and Genomics, Wayne State University, Detroit, MI, 48201.

5. Department of Obstetrics and Gynecology, Wayne State University, Detroit, MI 48201

6. Corresponding Author: douglasr@wayne.edu (office: 313-577-6688)

Running Title: Traumatic brain injury and long intron retention

Supplemental Figure 1: Gene ontology analysis of 374 unique genes which undergo intron retention 24 hours post-TBI. The output was filtered by FDR ≤ 0.05.

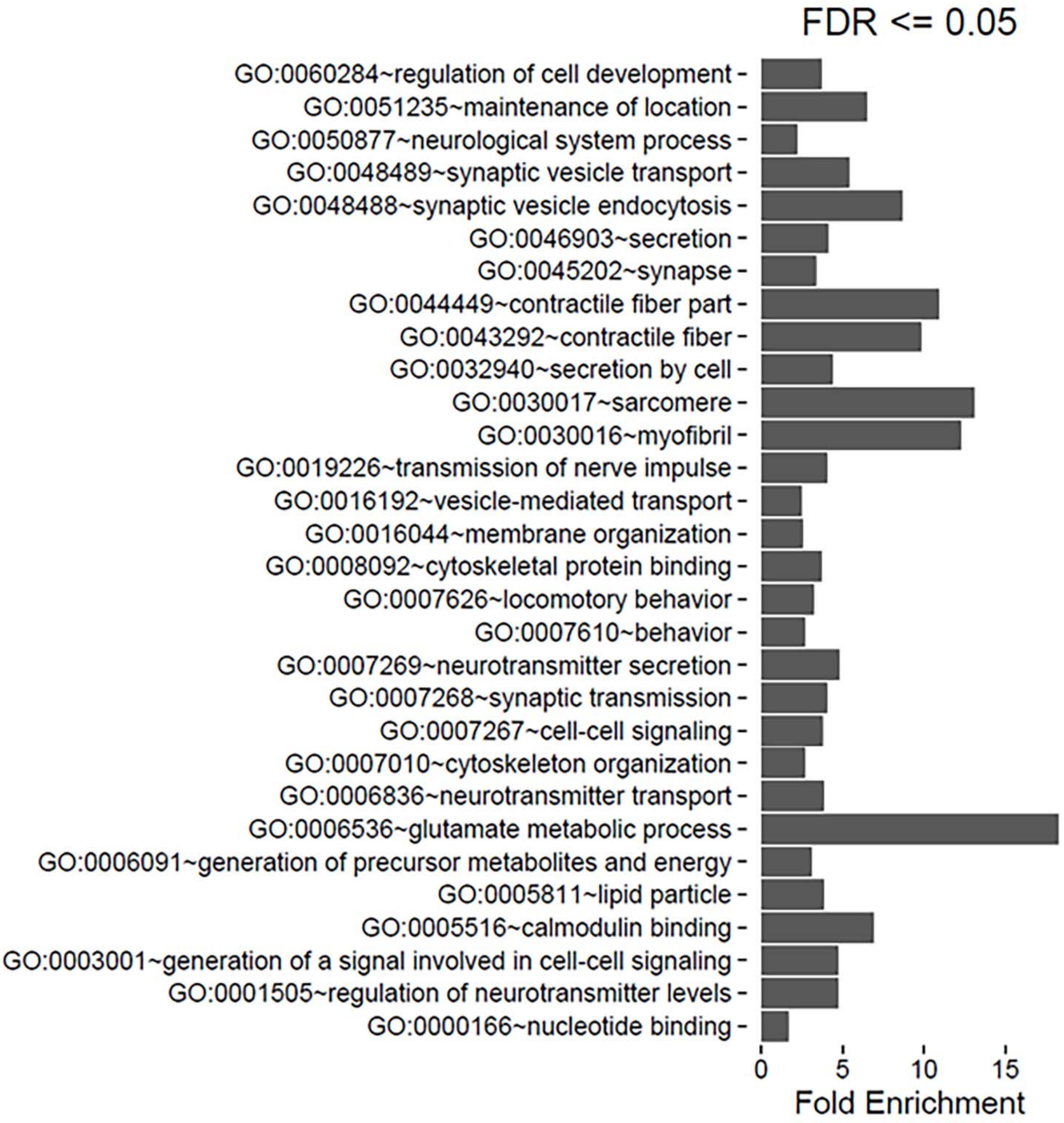

Supplementary Figure 2. Motif enrichment for 5'SS. 299 bps sequences were collected from intronic side of 5'SS for retained intron of length > 600 (N = 421/458). Motif enrichment analysis was run using reshuffled sequences as background (Parameters: Strand Handling = Only the given strand is processed; E-value threshold = 0.05; Max Motif Count = 10).

IUPAC	P-Value	E-Value	Motif (5'SS)	RNA Motif (Ray et al, 2013)	Gene
GTRAGT	1.4e-039	9.1e-035	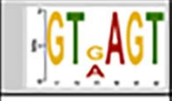	Not found	Not found
TAYATAY	3.5e-014	2.3e-009	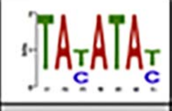	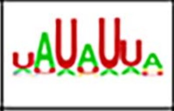	SHEP
TGTGTGKG	3.3e-013	2.2e-008	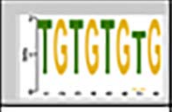	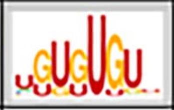	ARET/TDP43
CRCACAY	2.4e-011	1.6e-006	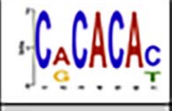	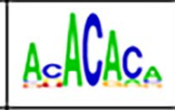	SM
BCGAAA	1.2e-009	7.8e-005	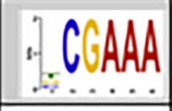	Not found	Not found
MTCGATT	1.6e-008	1.0e-003	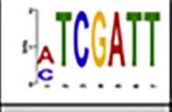	Not found	Not found
GGAAAA	2.5e-007	1.6e-002	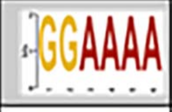	Not found	Not found
TTTRTTT	4.9e-007	3.2e-002	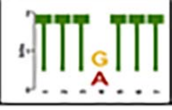	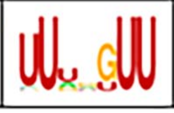	RBP9

**Parameters used in DREME; Strand Handling=Only the given strand is processed, E-value Threshold=0.05, Max Motif Count= 10

Supplementary Figure 3. Motif enrichment for 3'SS. 299 bps sequences were collected from intronic side of 5'SS for retained intron of length > 600 (N = 421/458). Motif enrichment analysis was run using reshuffled sequences as background (Parameters: Strand Handling = Only the given strand is processed; E-value threshold = 0.05; Max Motif Count = 10).

IUPAC	P-Value	E-Value	Motif (3'SS)	RNA Motif (Ray et al, 2013)	Gene
TTBCAG	3.2e-018	2.1e-013	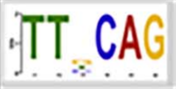	Not found	Not found
ATAYATWT	1.6e-012	1.1e-007	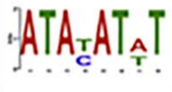	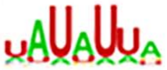	SHEP
ACTAATT	4.7e-008	3.0e-003	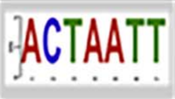	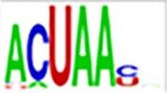	SF1
AHACAAA	5.3e-008	3.4e-003	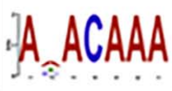	Not found	Not found
RCAGA	1.6e-007	1.0e-002	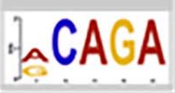	Not found	Not found
TTCKTTTT	1.8e-007	1.1e-002	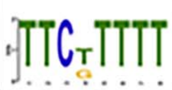	Not found	Not found
CACMCAC	4.9e-007	3.1e-002	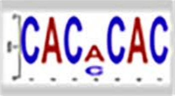	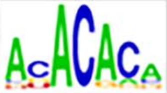	SM
AYATATGT	6.0e-007	3.8e-002	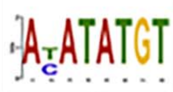	Not found	Not found

**Parameter; Strand Handling= Only the given strand is processed, E-value Threshold=0.05, Max Motif Count=10.

Supplemental Figure 4: Reads Per Kilo-base per Million (RPKM) in exons for control and dKDM4A-mutant 3rd instar larvae show complete knockdown of expression of dKDM4A / (Lysine (K)-Specific Demethylase .

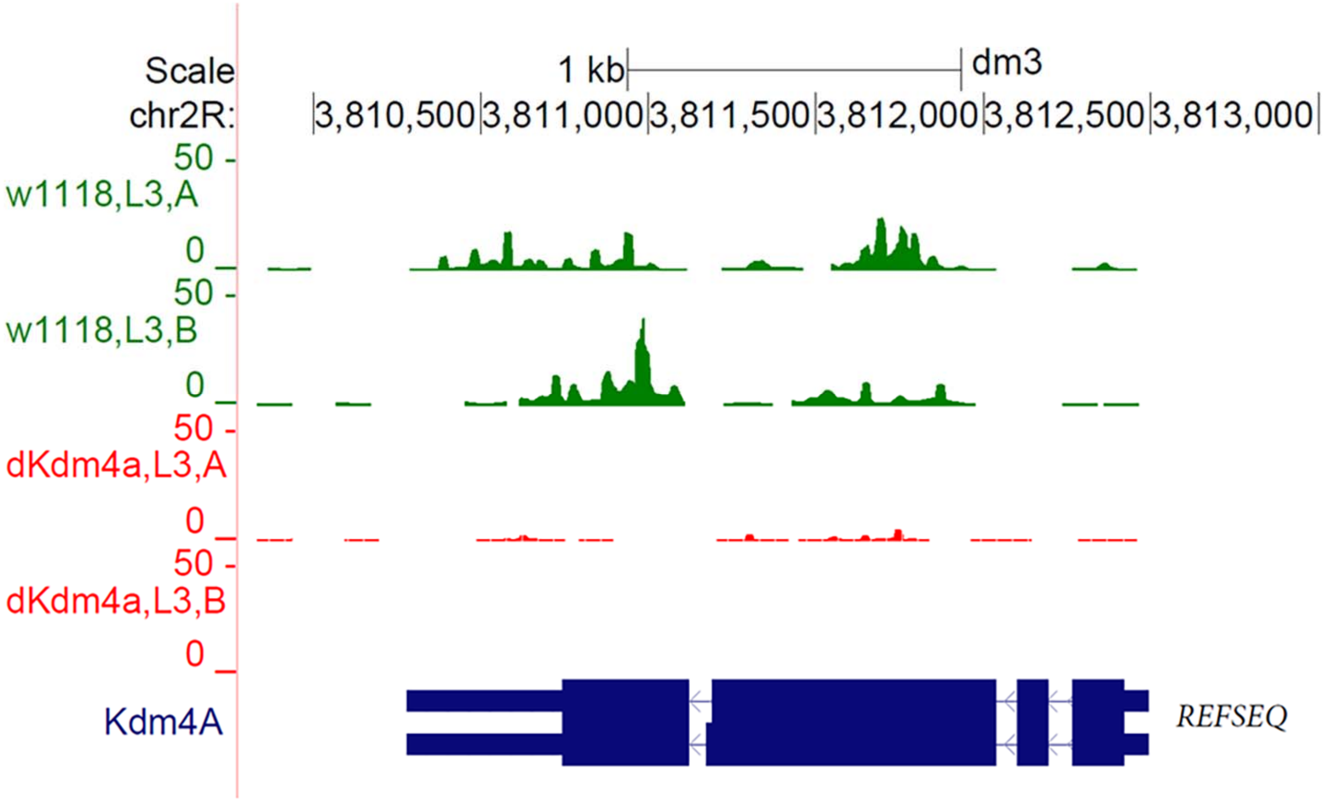

Supplemental Figure 5: Quantitative real-time PCR (qRT-PCR) validation of intron retention (RI) events in three genes. **A,** Eno1 mRNA shows 1.1-1.5-fold increase in RI relative to control. Two biological replicates were analyzed in triplicate using the indicated primers in a qPCR reaction (See Methods Section). **B,** Pyk mRNA shows a 7.0-26.0-fold increase in RI relative to control. **C,** StnA mRNA shows a 2.1-2.2-fold increase in RI relative to control. *, p-value < 0.05 (t-test, 2-tailed). **, p-value < 0.01 (t-test, 2-tailed). See **Supplementary Table 1** for oligo sequences and data analyses. We note that the fold-change in RI is an underestimate as unspliced mRNA is often subject to nonsense mediated decay and degraded.

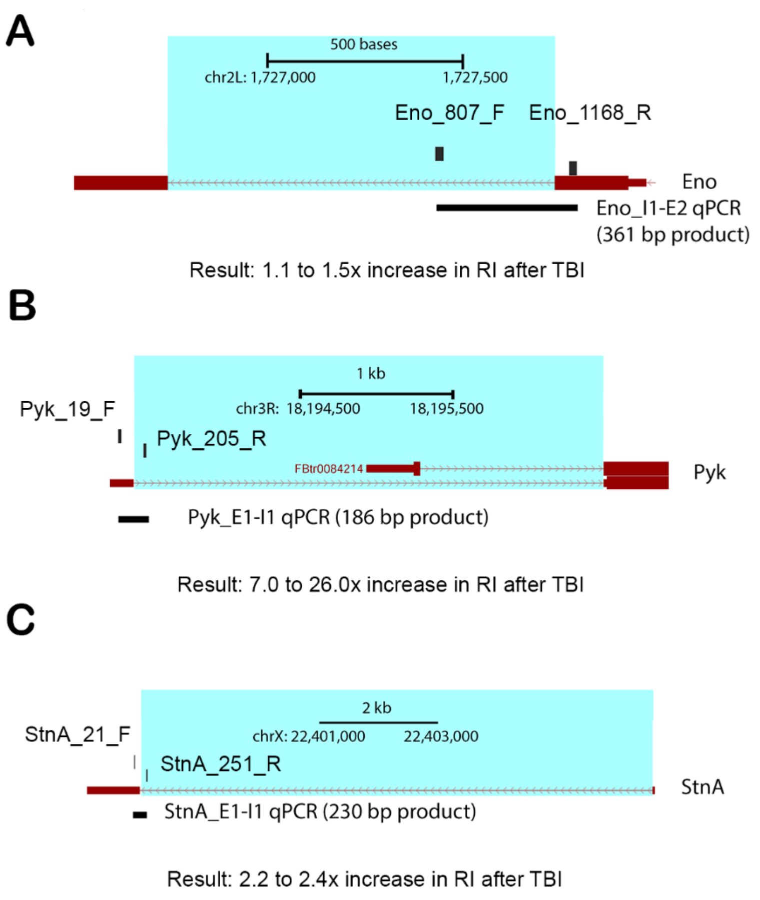

Supplement: Supplementary file 1 [file Image1.PDF]
